# Beyond surveillance: privacy, ethics, and regulations in face recognition technology

**DOI:** 10.3389/fdata.2024.1337465

**Published:** 2024-07-03

**Authors:** Xukang Wang, Ying Cheng Wu, Mengjie Zhou, Hongpeng Fu

**Affiliations:** ^1^Sage IT Consulting Group, Shanghai, China; ^2^School of Law, University of Washington, Seattle, WA, United States; ^3^Department of Computer Science, University of Bristol, Bristol, United Kingdom; ^4^Khoury College of Computer Science, Northeastern University, Seattle, WA, United States

**Keywords:** facial technology, technology and law, privacy, technology and regulations, ethical analysis

## Abstract

Facial recognition technology (FRT) has emerged as a powerful tool for public governance and security, but its rapid adoption has also raised significant concerns about privacy, civil liberties, and ethical implications. This paper critically examines the current rules and policies governing FRT, highlighting the tensions between state and corporate interests on one hand, and individual rights and ethical considerations on the other. The study also investigates international legal frameworks aimed at protecting individual rights and privacy, arguing that current legislative measures often fall short of robust scholarly standards and international human rights norms. The paper concludes with recommendations for developing principled and adaptable governance frameworks that harness the benefits of FRT while mitigating its risks and negative impacts, underscoring the importance of placing human rights and ethics at the center of regulating this transformative technology.

## 1 Introduction

In recent years, facial recognition technology (FRT) has emerged as a double-edged sword, offering significant benefits across various societal sectors while simultaneously presenting complex ethical, legal, and personal challenges (Shao et al., 2021). This technology, which identifies and verifies individuals by analyzing facial features from videos or images, has become increasingly integrated into daily life and institutional governance (Mantello et al., 2023). Its applications range from enhancing security protocols and consumer experiences to streamlining administrative processes, marking a notable improvement in operational efficiency (Shore, 2022). However, the rapid adoption of FRT in these areas raises important questions about individual privacy, data security, and ethical implications, necessitating a thorough academic examination that goes beyond superficial benefits (Palmiotto and González, 2023). Consequently, FRT has sparked legal controversies in many countries (Lai and Patrick Rau, 2021).

Although researchers began studying FRT in the 1950's and 1960's, progress was limited. However, since the 2000's, advancements in machine learning theory have significantly accelerated facial recognition research (Butt et al., 2023). FRT software based on traditional methods reached maturity and began to be used commercially in 2009. By 2013, FRT was widely employed in the commercial sector and had established a strong reputation (Zhong et al., 2021). This development caught the attention of several governments, who encouraged further research in the field (Yang et al., 2023).

This paper aims to contribute to the academic discourse surrounding FRT by critically analyzing its societal impacts, regulatory environment, and the delicate balance between collective security and individual liberties. Rather than merely summarizing current practices, the discussion will provide a critical analysis of the technology's implications, informed by historical, legal, and ethical scholarship. This involves a rigorous examination of international legal frameworks dedicated to protecting individual rights, specifically privacy, and how these principles intersect with the growing use of FRT.

Moreover, recognizing the transformative potential of FRT, this study adopts a multidimensional approach to capture diverse stakeholder perspectives. It examines the technology's adoption within state apparatuses, its reception among private citizens, and its broader societal ramifications, thereby addressing a gap in the literature regarding comprehensive, balanced analysis. By engaging with contemporary scholarly debates, the paper highlights the contested nature of FRT, illuminating the spectrum of academic thought on its ethical deployment and regulation.

In analyzing the intricate interplay between technological advancement, state oversight, and individual rights, this paper argues that the narrative surrounding FRT is multifaceted and complex. As such, any proposed or adopted regulatory mechanisms must be subject to scholarly scrutiny, ensuring they address not only the technology's practical aspects but also its broader societal, ethical, and legal implications. This approach underscores the necessity of a paradigm that respects human dignity, individual freedoms, and democratic values in the face of relentless technological progress.

The paper is structured as follows: Section 2 describes the methodology employed in this study, including the literature review, case study analysis, legal and regulatory framework assessment, and ethical and societal impact evaluation. Section 3 presents the results of the literature review, covering technical aspects and applications of FRT, legal and regulatory frameworks governing FRT use, and ethical and societal implications of FRT deployment. Section 4 investigates two case studies that illustrate the real-world implications of FRT and highlight key issues and challenges. Section 5 provides an overview of the legal and regulatory landscape governing FRT in the United States, focusing on government and private sector use. Section 6 offers a critical discussion of the key challenges and opportunities for developing a principled and rights-protective approach to FRT governance, addressing the need to balance utility and human rights, gaps in current regulatory frameworks, and the importance of inclusive and interdisciplinary collaboration. Finally, Section 7 concludes the paper by summarizing the key findings, offering recommendations for policymakers and stakeholders, and identifying areas for future research.

## 2 Methodology

This study employs a multi-method approach to examine the complex landscape of facial recognition technology and its implications for privacy, ethics, and regulation. By combining a comprehensive literature review, in-depth case study analysis, legal and regulatory framework assessment, and ethical and societal impact evaluation, we aim to provide a holistic understanding of the key issues surrounding FRT and propose a principled approach to its governance.

### 2.1 Literature review

To establish a solid foundation for our analysis, we conducted a comprehensive review of academic literature, legal documents, and policy reports related to FRT. The literature review focused on three main areas: (1) ethical implications of FRT use, including privacy, consent, bias, and discrimination; (2) current legal and regulatory frameworks governing FRT in the United States and internationally; and (3) the societal impact of FRT deployment, including security trade-offs, surveillance normalization, and democratic accountability.

The literature search was conducted using academic databases such as Google Scholar, Web of Science, and LexisNexis, as well as official government websites and policy repositories. Key search terms included “facial recognition technology,” “biometric privacy,” “FRT regulation,” “FRT ethics,” and “surveillance society.” The review encompassed a diverse range of sources, including peer-reviewed journal articles, conference proceedings, legal opinions, legislative documents, and policy briefs.

### 2.2 Case study analysis

To illustrate the real-world implications of FRT and highlight key issues and challenges, we selected two case studies for in-depth analysis. The first case study examines the legal controversy surrounding Clearview AI, a company that scraped billions of images from social media and other online sources to create a massive facial recognition database. This case raises critical questions about privacy violations, non-consensual data collection, and the lack of regulatory oversight in the private sector use of FRT.

The second case study focuses on the Transportation Security Administration's (TSA) pilot program for implementing FRT in U.S. airports. This case highlights issues of data protection, algorithmic bias, and the need for clear guidelines governing the collection, use, and storage of biometric data in public spaces.

For each case study, we analyzed primary sources, including legal complaints, court opinions, and government reports, as well as secondary sources such as media coverage and expert commentary. We assessed the key issues raised by each case, the legal and ethical implications, and the lessons learned for FRT governance.

### 2.3 Legal and regulatory framework analysis

To understand the current state of FRT regulation in the United States, we conducted a comprehensive analysis of federal, state, and local laws and policies governing the use of FRT by government agencies and private entities. This analysis included a review of relevant statutes, such as the Illinois Biometric Information Privacy Act, the California Consumer Privacy Act, and the proposed federal Commercial Facial Recognition Privacy Act.

We examined the scope and requirements of these laws, including provisions related to notice and consent, data protection, purpose limitation, and enforcement mechanisms. We also assessed the gaps and inconsistencies in the current regulatory landscape, highlighting the need for a more cohesive and comprehensive approach to FRT governance.

By combining these methodological approaches, literature review, case study analysis, and legal and regulatory framework assessment, we aim to provide a comprehensive understanding of the complex issues surrounding facial recognition technology. This multi-method approach allows us to identify key challenges, best practices, and recommendations for developing a principled, rights-protective approach to FRT governance.

## 3 Literature review

The academic literature on FRT spans multiple disciplines, including computer science, law, ethics, and social science. This review focuses on three main areas: (1) technical aspects and applications of FRT; (2) legal and regulatory frameworks governing FRT use; and (3) ethical and societal implications of FRT deployment.

### 3.1 Technical aspects and applications of FRT

FRT systems use computer algorithms to analyze and compare facial features for the purposes of identification or verification (Introna and Nissenbaum, 2010). The technology has advanced significantly in recent years, driven by developments in machine learning, particularly deep learning techniques such as convolutional neural networks (CNNs; Parkhi et al., 2015; Guo et al., 2016). CNNs have enabled FRT systems to achieve high accuracy rates on benchmark datasets, often surpassing human performance (Phillips et al., 2018).

FRT has a wide range of applications, including law enforcement, border control, access control, and commercial uses such as mobile phone authentication and targeted advertising (Gates, 2011; Jain et al., 2016). In the law enforcement context, FRT is used for tasks such as identifying suspects, tracking individuals across multiple cameras, and searching for missing persons (Klontz and Jain, 2013). Border control agencies use FRT for identity verification and screening purposes (Broeders, 2007). Commercial applications of FRT include face-based authentication for devices and services, as well as personalized marketing and customer tracking (Andrejevic, 2017).

### 3.2 Legal and regulatory frameworks governing FRT use

The legal and regulatory landscape governing FRT use varies widely across jurisdictions (Kugler, 2019). In the United States, there is no comprehensive federal law regulating FRT, although some states and cities have enacted their own biometric privacy laws (Acquisti et al., 2014). The most notable example is the Illinois Biometric Information Privacy Act, which requires companies to obtain informed consent before collecting biometric data and provides individuals with a private right of action for violations (Satariano, 2020).

At the federal level, the U.S. Government Accountability Office has called for the development of a comprehensive framework to regulate FRT use by government agencies (Government Accountability Office, 2020). The proposed Commercial Facial Recognition Privacy Act would prohibit commercial entities from using FRT to identify or track individuals without their affirmative consent (Commercial Facial Recognition Privacy Act, 2019).

Internationally, the European Union's General Data Protection Regulation classifies biometric data as a special category of personal data, subject to additional protections and restrictions (European Union, 2016). The GDPR requires explicit consent for the processing of biometric data and grants individuals the right to object to such processing (Veale et al., 2018). Other countries, such as China, have embraced FRT as a tool for public security and surveillance, with fewer restrictions on its use (Qiang, 2019).

### 3.3 Ethical and societal implications of FRT deployment

The widespread deployment of FRT raises significant ethical and societal concerns. One of the primary issues is the impact on privacy and individual autonomy (Brey, 2004; Andrejevic and Selwyn, 2020). The collection and use of biometric data without adequate safeguards or consent can infringe on individuals' right to control their personal information and can lead to a chilling effect on behavior (Rouvroy, 2015).

Studies have also highlighted the potential for bias and discrimination in FRT systems (Buolamwini and Gebru, 2018; Raji et al., 2020). Research has shown that some commercial FRT systems exhibit higher error rates for certain demographic groups, particularly people of color and women (Grother et al., 2019). This bias can lead to disproportionate impacts on marginalized communities, such as false arrests or denial of services (Garvie et al., 2016).

The use of FRT for surveillance purposes also raises concerns about the erosion of privacy in public spaces and the potential for abuse by government authorities (Hartzog, 2018; Lynch, 2020). The normalization of constant monitoring can have a chilling effect on free speech and association, undermining democratic values (Rouvroy, 2015).

Scholars have called for the development of ethical frameworks to guide the responsible use of FRT (Tene and Polonetsky, 2013; Floridi, 2018). These frameworks emphasize principles such as transparency, accountability, fairness, and respect for individual rights (Crawford and Schultz, 2014; Selbst and Barocas, 2018). Some researchers have proposed technical solutions to mitigate the risks of FRT, such as privacy-preserving algorithms and secure multiparty computation (Erkin et al., 2009).

This literature review highlights the complex technical, legal, and ethical dimensions of FRT. While the technology offers significant benefits, its deployment also poses risks to individual rights and societal values. Addressing these challenges requires a multidisciplinary approach that considers the perspectives of various stakeholders and balances the need for innovation with the protection of fundamental rights.

## 4 Case studies

### 4.1 Case study I: misuse of face recognition—Clearview legal controversy of AIs

To create a comprehensive biometric database, Clearview AI, an American technological innovation firm with its headquarters located in New York and launched in 2016, uses image scanners to automatically collect images of faces from social media and publicly available network platforms (Rezende, 2020). The business provides its services to both private businesses and law enforcement.

To utilize the facial recognition feature of Clearview AI, users need to take four primary actions (Bowyer, 2004): To digitally represent each face picture, (i) face photos from various websites are gathered and stored in a database; (ii) biometric identifiers are created; (iii) users are able to upload pictures and have them compared to the biometric identifiers stored in the database; and (iv) a series of comparison results are displayed, allowing users to view the source file of the identified photo. Interestingly, Clearview has over three billion face photographs in its collection—many of which are images of kids (Naga and Marri, 2023). Their services have been adopted by more than 600 law enforcement agencies in the United States, including prominent entities like the FBI, the Department of Homeland Security, and various state police departments (Buolamwini, 2018).

After the disturbances at the US Congress on January 6, 2021, state police departments in Florida and Alabama used facial search technology to identify persons implicated in the rioting. Clearview had a 26% increase in face search applications. Nonetheless, Clearview has encountered legal difficulties in a number of US states, including Vermont, New York, Illinois, and Virginia. Of these, at least three have been filed in Illinois alone. Macy's, a well-known shop, is one of Clearview's main clients, and it has been alleged that it has used facial recognition software. Furthermore, Clearview received explicit cease-and-desist letters from Twitter and Google prohibiting the gathering of face images on their networks. Clearview asserts that it has the authority to gather images that have been placed online despite these acts (Zhang et al., 2023).

Similar concern has been raised in certain foreign law enforcement agencies' native nations as a result of their use of Clearview's services. By using Clearview's services, the Swedish police department violated the “Criminal Data Act” and was fined SEK 2.5 million by the Swedish privacy protection agency IMY in February 2021 (Eneman et al., 2022). Between October 2019 and March 2020, the police department employed the Clearview face recognition app sporadically to find suspects and victims of crimes. However, according to IMY, there were multiple infractions of the “Criminal Data Act” with this activity. The Act states that genetic and biometric data may only be used for certain, clearly defined objectives in certain situations.

An investigation of Clearview was carried out in February 2021 by the personal information protection offices of British Columbia, Alberta, and the Office of the Privacy Commissioner of Canada (McSorley, 2021). As per the inquiry report, Clearview's facial recognition technology was found to have violated the standards of unified and appropriate purpose under the personal information protection legislation of Canada (McSorley, 2021). For “publicly available information,” the Canada Personal Information Protection and Electronic Documents Act waive the subject's consent requirements; however, face data gleaned through open websites, such as social media, is not covered by this exemption. Moreover, Clearview's actions violated the appropriate purpose requirement of the Act, which still applies even with valid consent. The company inappropriately collected and used images in ways unrelated to the original purpose for which the photos were uploaded, and it retained these photos indefinitely, posing a significant risk to individuals' personal interests, such as being used against the uploader in subsequent prosecutions. Additionally, Clearview's indiscriminate collection of face photos from websites was deemed an unreasonable information-gathering method.

### 4.2 Case study II: the implementation of facial recognition technology in U.S. airports

In recent developments, the Transportation Security Administration initiated a pilot project to assess the implementation of facial recognition technology across several U.S. airports (Boudreaux et al., 2022). This program involved passengers using an automated system to verify their identities by scanning their ID and matching it with their facial image, without the need for direct interaction with TSA officers.

The technology is currently being tested in 16 airports including major hubs like Atlanta, Boston, Dallas, and Miami. Travelers use a device to scan their driver's license or passport, after which they are required to look into a camera. The system then compares the live image to the photo ID. While a TSA officer oversees the process, the interaction is minimal.

The pilot program is voluntary, but it has raised significant concerns among privacy advocates and some elected officials. Critics argue that the increased use of biometric surveillance by the government poses risks to civil liberties and privacy rights (Carter, 2018). Furthermore, concerns about the potential bias in FRT, particularly in accurately recognizing faces of minorities, and the security of biometric data against hacking, have been highlighted (Palmer, 2020).

Critics express concern about the future of data storage and the fairness of putting the burden of opting out on passengers (Garvie, 2019). Jeramie Scott from the Electronic Privacy Information Center emphasizes the need for an independent audit to verify the technology's impartiality and the immediate deletion of images (Scott, 2016, 2017).

The TSA, however, asserts that the goal is to enhance identity verification accuracy without compromising checkpoint efficiency. They claim that the images are not compiled into a database, with certain data being retained for assessment purposes only and deleted after 24 months (Khan and Efthymiou, 2021). TSA also notes that the technology provides passengers with control over its use and that its algorithm shows no discernible bias (Khan and Efthymiou, 2021).

This case raises critical questions about the balance between technological advancement in security and the protection of individual privacy rights. It also underscores the increasing integration of biometric technology in everyday life and the challenges in regulating and overseeing its use in public domains.

## 5 Legal and regulatory framework review

### 5.1 Legal regulation of face recognition in the United States

The United States has been at the forefront of face recognition technology and its legislative efforts (Chen and Wang, 2023). However, the legal regulation of face recognition in the country takes different paths based on the users of this technology, resulting in differentiated regulatory approaches. Notably, the legal regulation for the use of face recognition by government departments and its use by non-governmental organizations is legislated and regulated separately, with distinct methods and value orientations guiding the regulations.

### 5.2 Legal regulations on the use of face recognition by government departments

There are three primary categories of legal regulations about the use of face recognition by government departments, based on current and proposed legislation in the United States: (i) the regime of prohibited use, (ii) the regime of special permission to use, and (iii) the regime of discretionary use (Garvie, 2016). San Francisco, California, was the first city in the nation to implement the restricted use policy, which is now gaining popularity (Conger et al., 2019). While government agencies are permitted to utilize face recognition technology without explicit law under the discretionary use regime, the special licensing system is now in the public proposal stage.

In May 2019, the San Francisco Board of Supervisors passed the “Stop Secret Surveillance Ordinance,” prohibiting the use of facial recognition technology by any government agency, including the police department (Conger et al., 2019). In addition, the act mandates that city departments seek approval from the Board of Supervisors before disclosing any technologies they currently or plan to utilize for monitoring, as well as outlining their privacy policies (Conger et al., 2019). San Francisco has become the first city in the world to outlaw face recognition technology, as this ordinance does not apply to the use of face recognition technology for personal, commercial, or federal government purposes.

Comparably, the city of Somerville, Massachusetts passed the “Banning the Usage of Facial Technology Surveillance in Somerville” law in June 2019 (Nieves, 2021). This law prohibits the city's government agencies, including the courts, from obtaining, retaining, using, or accessing facial surveillance systems or the personal data they may have collected. Any data that is found needs to be erased right away. The right to file a lawsuit in any municipal court with jurisdiction is granted to victims of government departments' illegal use of facial surveillance systems and collection of personal facial information. These victims may seek compensation for their actual losses, with liquidated damages not to exceed $1,000 or $100 per offense, whichever is greater. The “Oakland Municipal Code,” Chapter 9.64, was amended by the city of Oakland in California in July 2019 (Young et al., 2019). As a result, no department within the city is allowed to obtain, keep, request, use, or obtain face recognition software.

There is strong opposition to the use of facial recognition technology for public surveillance in many other states in the US, in addition to the legislative steps done in San Francisco, Somerville, and Oakland. A few opponents have gone so far as to create a special website where they advocate for the outright prohibition of face recognition technology and gather signatures on petitions. They contend that facial recognition should be outlawed entirely and that merely regulating it is insufficient.

Additionally, the U.S. Senate was debating the “Ethical Use of Face Recognition Act (Draft)” in February 2020 (Wang, 2020). This proposed act asks Congress to establish a face recognition committee whose job it is to provide standards for the moral use of facial recognition technology. The draft statute forbids any government departments from implementing face recognition technology or using it to gather personal information until these guidelines are formally issued. Furthermore, it expressly prohibits law enforcement from identifying particular people using face recognition technology without first obtaining an arrest warrant (Madzou and Louradour, 2020; Shao et al., 2021; Yang et al., 2023).

### 5.3 Legal regulations on the use of face recognition by non-governmental organizations

The legal regulation of face recognition technology used by non-governmental organizations in the United States primarily focuses on treating face information as a form of biological information (Monajemi, 2017). This regulation can be categorized into two distinct paths: one follows a high-intensity or special regulatory path that imposes stricter measures compared to the protection of general personal information, while the other adopts an ordinary regulatory path with similar levels of protection as general personal information (Almeida et al., 2022).

### 5.4 Special regulatory

The special regulatory approach for the use of face recognition by non-governmental organizations is exemplified by the Illinois “Biological Information Privacy Act” and the “Commercial Face Recognition Privacy Act” currently being considered by the U.S. Congress (Zhou, 2020). The Biometric Information Privacy Act (BIPA), enacted in Illinois in 2008, stands as the first state-level law in the United States to safeguard personal biological information (Buresh, 2021).

BIPA differentiates between “biometric identifiers” and “biometric information.” “Biometric identifiers” encompass specific attributes such as retinal or iris scans, fingerprints, voiceprints, or scans of hand or facial geometry. “Biometric information” pertains to any data derived from a biometric identifier, which is utilized to identify an individual.

Human face naturally falls under the category of “biometric identifier” and thus qualifies as “biological information” under BIPA. It is essential to note that BIPA solely regulates private entities, which include individuals, partnerships, companies, etc., but specifically excludes government agencies and courts.

According to BIPA, private entities are required to furnish prior notice and obtain explicit consent from individuals before collecting their biometric information. Both the notice and consent must be provided in writing, with the consent being “informed written consent.” Furthermore, BIPA prohibits any private entity in possession of biometric identifiers or biometric information from engaging in activities such as selling, leasing, trading, or profiting from an individual's or customer's biometric identifier or biometric information (Beltrán and Calvo, 2023).

Under BIPA, private entities that possess biometric identifiers or biometric information are subject to two key security protection requirements:

1. **Standard of reasonable care**. Private entities must adhere to a “standard of reasonable care within the private entity profession.” This means that the level of care required may vary depending on the specific industry. The determination of what constitutes a “reasonable” standard of care is not based on intuition but often relies on jury verdicts or judicial decisions.
2. **Inclusion of biometric information at least equivalent to the protection of “confidential and sensitive information.”** Private entities are obligated to safeguard biometric identifiers and biometric information with a level of protection that is at least equivalent to that provided for “confidential and sensitive information.” This ensures that biometric data receives the same or higher level of protection as other sensitive data. BIPA also grants victims the right to take legal action against private entities that breach any provisions of the law. In case of a successful legal claim, the victim may be eligible to receive either liquidated damages or actual damages, depending on whichever amount is greater, for each violation committed by the defendant.

The Illinois Supreme Court rendered a major decision in the Rosenbach v. Six Flags Entertainment Corp case, holding that plaintiffs are entitled to damages under BIPA without having to prove actual damages (Stepney, 2019). 1. The court concluded that the defendant had violated BIPA by emphasizing the importance of biological information's inalterability. Because “when a private entity fails to comply with statutory procedures, the right of individuals to maintain their biological information privacy disappears,” the plaintiff's injury was considered “real and significant.” Premature to seek liquidated damages and injunctive remedies until after real losses have occurred would thus be in opposition to the goal of BIPA, which is to stop and discourage the unlawful gathering and use of personal biological information.

On another front, the “Commercial Facial Recognition Privacy Act of 2019” has undergone multiple reviews by the US Congress (Gies et al., 2020). Its primary objective is to prohibit commercial organizations from using face recognition technology without obtaining affirmative consent from end users for identification or tracking purposes. The Act specifies that data processors cannot use facial recognition technology to collect facial recognition data unless explicit consent is obtained from the end user, accompanied by a clear and unambiguous notice that informs the end users about the face recognition technology's functions, limitations, and how to obtain more information from data processors. Additionally, the use of facial recognition technology to discriminate against users is also deemed illegal under this Act.

### 5.5 General regulation

The California Consumer Privacy Act (CCPA) provides a general legislative framework for non-governmental companies using face recognition technology (Baik, 2020). Biological information, which includes facial information, is regulated as personal information under the CCPA. If a company's gross yearly income surpasses $25 million or if it gathers personal data from more than 50,000 customers each year, it must abide by the CCPA. Additionally, businesses that collect personal data from more than 137 people every day are subject to the CCPA.

Since many facial recognition systems satisfy the CCPA's requirements, their operators must abide by the laws laid forth in the act. Regulations comparable to those governing the collecting of general personal information also apply to the gathering of personal biological information, including facial data, under the CCPA. It's crucial to remember, though, that in comparison to certain other legal frameworks, the CCPA's rules on personal biological information are comparatively laxer.

## 6 Discussion

The rapid advancement and deployment of FRT have brought to the fore a complex array of ethical, legal, and societal implications. As our case studies and regulatory analysis have shown, the current landscape of FRT use is characterized by a patchwork of laws, a lack of comprehensive oversight, and inadequate protections for individual rights and privacy. This section discusses the key challenges and opportunities for developing a more principled and rights-protective approach to FRT governance.

### 6.1 Balancing utility and human rights

One of the central challenges in regulating FRT is striking the right balance between the technology's potential benefits and the need to safeguard fundamental human rights. FRT offers significant utility in various domains, from enhancing security and streamlining identification processes to enabling personalized services and experiences. However, as our case studies illustrate, the deployment of FRT can also lead to serious violations of privacy, consent, and non-discrimination when proper safeguards and oversight are lacking.

The ascent of facial recognition technology brings to the fore significant ethical quandaries, especially regarding individual privacy and autonomy. At the individual level, FRT challenges conventional conceptions of privacy, particularly the notion of “privacy in public”—an individual's right to anonymity in public spaces (Meden et al., 2023). FRT effectively nullifies anonymity, as facial features, unlike traditional identifiers such as passwords, cannot be easily altered or concealed without attracting scrutiny. This persistent visibility raises profound ethical questions about consent and the commodification of personal identity.

The current scholarly debate underscores the inadequacy of implied consent in public spaces, advocating instead for explicit, informed consent that recognizes the sensitivity of facial data (Zennayi et al., 2023). The ethical conundrum emerges from the lack of viable alternatives for individuals unwilling to surrender their biometric data, often necessitating withdrawal from public or societal utilities—a form of coercive consent that contravenes ethical norms (Beltrán and Calvo, 2023).

Furthermore, FRT's deployment often occurs without the explicit informed consent of those subjected to it, infringing upon the ethical principle of respect for persons' autonomy. Individuals are frequently unaware of when, how, and for what purpose their biometric data is being collected and analyzed (Vijaya Kumar and Mathivanan, 2023). This covert data harvesting not only breaches personal privacy but also engenders power asymmetries between data subjects and the entities wielding the technology, be they governmental or corporate.

The technology's operation—analyzing, quantifying, and cataloging human faces—arguably reduces individuals to mere data points within vast informational networks. This commodification of personal identity underscores concerns about dehumanization and potential abuses of power (Beltrán and Calvo, 2023). The efficiency benefits touted by FRT proponents must be weighed against these profound ethical compromises.

To navigate this complex landscape, current academic discourse advocates for a more person-centric approach to technology assessment. This approach emphasizes individuals' moral and legal entitlement to privacy and the imperative of maintaining human dignity in the face of technological advancement (Bingley et al., 2023a,b; Del Giudice et al., 2023). It calls for FRT governance frameworks that prioritize individual autonomy, meaningful consent, and the protection of “privacy in public.”

Striking the right balance between FRT's utility and the protection of human rights will require a multi-stakeholder, adaptive approach to governance. Policymakers, developers, and deployers of FRT must engage in ongoing dialogue with ethicists, legal experts, civil society, and impacted communities to ensure that the technology is developed and used in ways that respect individual rights and societal values. This includes implementing robust transparency and accountability measures, as well as providing meaningful options for individuals to opt-out of FRT processing.

Ultimately, the goal should be to harness the benefits of FRT while mitigating its risks and negative impacts. By centering human rights and ethics in the governance of FRT, we can work toward a future in which the technology serves the public good without compromising fundamental rights and freedoms. This will require not only technical and legal safeguards, but also a cultural shift toward greater valuation of privacy and individual autonomy in an increasingly digitized world.

### 6.2 Gaps and challenges in current regulatory frameworks

Our analysis of legal frameworks governing FRT reveals significant gaps and inconsistencies in the current regulatory landscape. At the federal level, there is no comprehensive law addressing the unique risks and challenges posed by FRT, leaving a patchwork of sector-specific and state-level regulations to fill the void (Garvie, 2019). While some states, such as Illinois and California, have enacted biometric privacy laws that provide important protections, the lack of a uniform federal standard creates uncertainty and uneven safeguards for individuals across the country (Scott, 2016).

Moreover, existing privacy laws and regulations, such as the European Union's General Data Protection Regulation (GDPR), may not fully capture the nuances and complexities of FRT (Khan and Efthymiou, 2021). For example, the GDPR's provisions on consent and data minimization, while important, may not adequately address the challenges of meaningful consent and purpose limitation in the context of FRT deployments in public spaces or for surveillance purposes (Garvie, 2016).

Addressing these regulatory gaps and challenges will require a concerted effort by policymakers, industry stakeholders, civil society organizations, and academic experts to develop a more comprehensive and harmonized framework for FRT governance. This framework should be grounded in human rights principles, such as necessity, proportionality, and non-discrimination, while also providing clear guidance on issues such as consent, transparency, accountability, and redress (Almeida et al., 2022; Naga and Marri, 2023).

### 6.3 Toward a principled regulatory approach

To move toward a more principled and rights-protective approach to FRT regulation, we propose the following key elements.

#### 6.3.1 Data minimization and purpose limitation

FRT regulations should require that the collection and use of biometric data be limited to what is necessary and proportionate for specific, legitimate purposes. This means prohibiting the indiscriminate or mass collection of facial biometrics, and ensuring that FRT systems are designed to minimize the amount of data collected and the duration of its retention (Garvie et al., 2016). Purpose limitation provisions should restrict the use of collected data to the original purposes for which it was obtained, and prohibit secondary uses without explicit consent or legal authorization.

#### 6.3.2 Transparency and informed consent

Individuals should have a right to know when and how their biometric data is being collected and used, and to provide meaningful consent for such practices. FRT regulations should mandate clear and conspicuous notice about the deployment of FRT systems, including information about the purposes of data collection, the entities involved, and the rights of individuals (Garvie, 2019). Where possible, individuals should be given the opportunity to opt-in or opt-out of FRT data collection and use. In contexts where individual consent may not be feasible, such as in public spaces, transparency measures should still be required to ensure public awareness and accountability.

#### 6.3.3 Ongoing oversight and auditing

Given the rapid pace of technological change and the evolving nature of FRT risks and harms, it is critical that any regulatory framework includes provisions for ongoing oversight and auditing of FRT systems and practices (Almeida et al., 2022; Naga and Marri, 2023). This could include mandatory impact assessments, regular audits by independent third parties, and continuous monitoring for accuracy, bias, and misuse. Oversight mechanisms should be transparent, accountable to the public, and empowered to enforce compliance and impose penalties for violations.

### 6.4 Aligning with international human rights frameworks

In developing a principled approach to FRT regulation, it is important to align with existing international human rights frameworks and standards. The Universal Declaration of Human Rights, the International Covenant on Civil and Political Rights, and other human rights instruments provide a foundation for protecting privacy, dignity, and non-discrimination in the context of emerging technologies (Chen and Wang, 2023).

Aligning FRT regulations with these international frameworks can help ensure consistency and interoperability across jurisdictions, facilitating cross-border data flows and cooperation in addressing transnational challenges. It can also provide a common language and set of principles for engaging in multi-stakeholder dialogue and collaboration on FRT governance issues (Naga and Marri, 2023).

### 6.5 The need for inclusive and interdisciplinary collaboration

Developing effective and legitimate FRT governance frameworks will require ongoing collaboration among a diverse range of stakeholders, including policymakers, industry leaders, civil society organizations, academic researchers, and affected communities. Inclusive and interdisciplinary collaboration can help ensure that multiple perspectives and expertise are brought to bear on the complex challenges of FRT, and that the resulting frameworks are informed by the lived experiences of those most impacted by the technology (Monajemi, 2017; Madzou and Louradour, 2020).

This collaborative approach should prioritize the voices and interests of marginalized and vulnerable communities, who may face disproportionate risks and harms from FRT deployments. It should also involve cross-disciplinary dialogue and knowledge-sharing, bringing together insights from computer science, law, ethics, social science, and other relevant fields to develop holistic and contextually grounded governance strategies (Naga and Marri, 2023).

## 7 Conclusion

The widespread adoption of FRT has brought to light a complex web of ethical, legal, and societal implications that necessitate a principled and proactive approach to governance. Our analysis reveals that the current landscape of FRT use is marked by a fragmented legal framework, insufficient oversight, and inadequate safeguards for individual rights and privacy. The case studies discussed in this paper serve as poignant examples of the regulatory gaps and ethical challenges surrounding FRT deployment, underscoring the pressing need for more comprehensive and harmonized legal frameworks.

At the heart of developing effective FRT governance is the need to strike a careful balance between the technology's potential benefits and the imperative to protect fundamental human rights. This requires a shift from a narrow focus on technical capabilities and efficiency gains to a more comprehensive consideration of the ethical and societal consequences of FRT use. By prioritizing principles such as transparency, accountability, data minimization, and informed consent, policymakers and stakeholders can work toward crafting governance frameworks that promote public trust and safeguard individual dignity.

However, the road ahead is fraught with challenges. The rapid pace of technological change, the transnational nature of data flows, and the competing interests of stakeholders complicate the development of coherent and adaptable regulatory approaches. Overcoming these obstacles will require sustained multi-stakeholder collaboration, drawing on the expertise and perspectives of policymakers, industry leaders, civil society organizations, academic researchers, and affected communities.

Furthermore, as the paper has emphasized, the development of principled FRT governance cannot be confined to national borders. In an increasingly interconnected world, it is essential to align domestic regulations with international human rights frameworks and standards, fostering cross-border cooperation and ensuring consistent protections for individuals across jurisdictions.

Ultimately, the way forward lies in embracing a proactive, inclusive, and ethically grounded approach to FRT governance. By placing human rights and democratic values at the core of the development and deployment of this transformative technology, we can work toward a future in which the benefits of FRT are harnessed for the greater good, while its risks and negative impacts are effectively mitigated. This will require not only technical and legal safeguards but also a fundamental shift in how we understand the relationship between technology, society, and individual autonomy.

As we study the complex FRT regulation, it is crucial to keep in mind the fundamental principles that should guide our efforts. By prioritizing transparency, accountability, and respect for human dignity, we can chart a path toward a more equitable and sustainable future—one in which the power of technology is harnessed to uplift, rather than undermine, the essential values that define us as a society.

## Ethics statement

Ethical approval was not required for the studies involving humans in accordance with the local legislation and institutional requirements. Written informed consent was obtained from the participants for participation in the study and for the publication of any identifying images or information included in the article.

## Author contributions

XW: Investigation, Writing – review & editing, Data curation, Formal analysis, Writing – original draft. YW: Investigation, Writing – original draft, Methodology, Resources, Writing – review & editing. MZ: Formal analysis, Investigation, Methodology, Writing – review & editing. HF: Investigation, Writing – review & editing, Project administration, Resources.
